# Could baseline health-related quality of life (QoL) predict overall survival in metastatic colorectal cancer? The results of the GERCOR OPTIMOX 1 study

**DOI:** 10.1186/1477-7525-12-69

**Published:** 2014-05-13

**Authors:** Momar Diouf, Benoist Chibaudel, Thomas Filleron, Christophe Tournigand, Marine Hug de Larauze, Marie-Line Garcia-Larnicol, Sarah Dumont, Christophe Louvet, Nathalie Perez-Staub, Alexandra Hadengue, Aimery de Gramont, Franck Bonnetain

**Affiliations:** 1 Direction de la Recherche Clinique et de l’Innovation, Centre Hospitalier Universitaire Amiens, Nord, 1, Place Victor Pauchet, F-80054, Amiens, Cedex, France; 2 Methodology and quality of life in oncology Unit, EA 3181 CHU Besançon and the Qualité de Vie et Cancer clinical research platform, Dijon, France; 3 Department of medical oncology, Hôpital Saint-Antoine, Assistance Publique des Hôpitaux de Paris, UPMC Paris VI, Paris, France; 4 Oncology Multidisciplinary Research Group (GERCOR), Paris, France; 5 Biostatistics Unit, Claudius Régaud Institute, Toulouse, France; 6 Department of medical oncology, Hôpital Henri Mondor, Assistance Publique des Hôpitaux de Paris, Créteil, Paris, France; 7 Department of medical oncology, Institut Mutualiste Montsouris, Paris, France

## Abstract

**Background:**

Health-related quality of life (QoL) has prognostic value in many cancers. A recent study found that the performance of prognostic systems for metastatic colorectal cancer (mCRC) were improvable. We evaluated the independent prognostic value of QoL for overall survival (OS) and its ability to improve two prognostic systems’performance (Köhne and GERCOR models) for patients with mCRC.

**Methods:**

The EQ-5D questionnaire was self-completed before randomization in the OPTIMOX1, a phase III trial comparing two strategies of FOLFOX chemotherapy which included 620 previously untreated mCRC patients recruited from January 2000 to June 2002 from 56 institutions in five countries. The improvement in models’ performance (after addition of QoL) was studied with Harrell’s C-index and the net reclassification improvement.

**Results:**

Of the 620 patients, 249 (40%) completed QoL datasets**.** The Köhne model could be improved by LDH, mobility and pain/discomfort; the C-index rose from 0.54 to 0.67. The associated NRI for 12-month death was 0.23 [0.05; 0.46]. Mobility and pain/discomfort could be added to the GERCOR model: the C-index varied from 0.63 to 0.68. The NRI for 12 months death was 0.35 [0.12; 0.44].

**Conclusions:**

Mobility and pain dimensions of EQ5D are independent prognostic factors and could be useful for staging and treatment assignment of mCRC patients. Presented at the 2011 ASCO Annual Meeting (#3632).

## Background

Colorectal cancer (CRC) is the third most diagnosed cancer in men and the second most diagnosed in women, with over 1.2 million new cases and 608 700 deaths worldwide in 2008 [1]. About up to half (20% to 50%) of CRC patients will develop metastases during the course of their disease [2] and approximately 35% are diagnosed with synchronous metastases [2,3]. Standard treatments for metastatic CRC (mCRC) are based on chemotherapy.

As is the case for many cancers, CRC staging is essential for optimal patient management. Accurate prognostication facilitates both therapeutic decisions and stratification in randomized clinical trials of cancer treatments. In CRC, the well-known TNM staging system is predominantly used [4]. In mCRC, two validated prognostic classification systems can be applied: Köhne prognostic index [5] for patients receiving front-line fluoropyrimidine mono-chemotherapy and GERCOR (Groupe Coopérateur Multidisciplinaire en Oncologie) prognostic index [6] for patients with oxaliplatin-based or irinotecan-based regimens. However, the models’ ability to discriminate between patients on the basis of their prognosis (as measured by the C-index [7]) is still relatively modest. Thus, improvement of these prognostic indicators is required [6].

In palliative care patients, the prognostic value of health-related quality of life (QoL) has been demonstrated for several types of cancer [8-10]. For mCRC patients, QoL is known to be an independent prognostic factor for overall survival (OS) [8,11]. Hence, QoL is a candidate for the improvement of existing prognostic indices. Given that QoL is a multidimensional concept, there is a need to identify the QoL dimensions associated with OS for each specific type of cancer. The results of a recent study showed that social functioning (as measured with the EORTC QLQ-C30 tool) is an independent prognostic factor for survival in mCRC patients [12]. The objective of the present study was to assess the independent prognostic value of QoL in mCRC and evaluate its ability to improve the Köhne and GERCOR prognostic indices.

## Methods

### Patients

Individual patient data from the OPTIMOX1 phase III trial were analysed. The 620 evaluable patients from OPTIMOX1 were recruited from January 2000 to June 2002 from 56 institutions in five countries. In this trial, the oxaliplatin stop-and-go strategy proved to be as good as a continuous oxaliplatin-based chemotherapy strategy in previously untreated mCRC patients. The trial's inclusion and exclusion criteria are detailed elsewhere [13].

### Quality of life assessment

Quality of life was self-reported by the patient using the generic EQ-5D questionnaire (also known as EuroQol) [14], which has five dimensions (mobility, self-care, usual activities, pain/discomfort and anxiety/depression) rated as one of three levels ("no problems",”some problems" and ”extreme problems", coded as 1, 2 and 3, respectively). The EQ-5D also includes a 100-centimetre visual analogue scale (VAS) for the self-assessment of overall health (0 = worst possible score; 100 = best possible score).

### The GERCOR and Köhne prognostic indices

The Köhne prognostic index [5] comprises four variables: performance status (PS), number of metastatic sites, alkaline phosphatase (ALP) level and white blood cell (WBC) count. The GERCOR prognostic index [6] is based on two variables: PS and serum lactate dehydrogenase (LDH) level. Patients are classified into three risk groups (low, intermediate and high) in both models.

### Statistical analysis

Demographic and clinical characteristics were summarized as frequency and percentage. In order to check whether selection bias was present, the patients’clinical characteristics were compared (with chi-squared test or Fisher's exact test) as a function of the available QoL data at baseline.

Overall survival was defined as the time from randomization to death (regardless of the cause) or last follow-up (censored data). All randomized patients with complete QoL data were included in the statistical analysis.

Univariate and multivariate analysis were performed using Cox proportional hazards modelling, with calculation of the hazard ratio (HR) and the corresponding 95% two-sided confidence intervals (95%CI).

In order to evaluate the independent prognostic value of QoL, we built two multivariate models with backward selection. The first model included all demographic and clinical variables associated with OS (p<0.1) in univariate analysis. The second model included demographic, clinical and QoL variables with p<0.1 in univariate analysis.

Improvements in the prognostic index was evaluated by adding clinical variables (other than those used to build the prognostic index) and QoL variables (with p<0.1 in univariate analysis) to a model with backward selection (Köhne or GERCOR index being forced in the model). Patients with available QoL data for whom Köhne and GERCOR indices could be calculated were considered for prognostic systems’ improvement.

The models were compared by calculating the Schemper statistic [15] and Harrell’s C index [7]. The Schemper statistic is equivalent to R^2^ in linear regression and quantifies the proportion of the survival variability that is explained by the model. Briefly, the higher the Schemper statistic is, the more accurate the OS predictions would be. Harrell’s C index estimates discriminate capability, i.e. the ability to distinguish between high-risk and low-risk patients. The C-index varies from 0.5 (no discrimination) to 1 (perfect discrimination). Optimism-corrected C-index was calculated using 200 bootstrap replications.

Category-free net reclassification improvement [16] (NRI) was also calculated at various moments (12, 24 and 36 months), in order to evaluate the additional utility of QoL domains and other clinical factors. NRI quantifies”the correctness of upward and downward reclassification or movement of predicted probabilities as a result of adding a new marker”. The confidence interval for NRI was calculated using the percentiles of 1000 bootstrap replications.

We also performed a sensitivity analysis using the multiple-imputation technique [17,18] (with 10 replications) for missing QoL data. The choice of 10 replications was prompted by the large amount of missing QoL data in the trial (60%). In line with Van Buuren’s method [19], the demographic and clinical variables initially included in the final complete-data model, those associated with the lack of QoL data and those strongly associated with OS (albeit absent from the final model) were used as predictors for the imputation of missing QoL data using a logistic regression model (QoL coded as 2–3 vs. 1). Multiple imputation with 10 replications (of the original database) consisted in creating 10 plausible values for each missing data and thus generating 10 new complete databases. For each of the new databases, a standard analysis was performed and the results were combined into a single estimation of the parameter of interest, while taking account of the uncertainty of the imputation technique [20]. Variables selected more than 5 times out of 10 replications were included in the multivariate model after multiple imputations.

Since there was no within-imputation variance according to the Schemper statistic, the pooled estimate was presented as the median [range] [20].

Construction of the a modified prognostic index was based on linear transformation as follows: The regression coefficient for each variable selected in the final multivariate complete case Cox model was divided by the lowest coefficient and rounded to the nearest integer [21]. The sum of these integers is the maximum score (M) for the modified index; hence the new score varied from zero to M. According to the score, the modified prognostic index was then arbitrary divided into three risk groups: good prognostic, intermediate prognostic and poor prognostic.

Survival distributions were estimated using the Kaplan-Meier method [22] and compared with the log-rank test.

All statistical analyses were carried out using SAS® software (version 9.2, SAS Institute Inc., Cary, NC) and R.2.12.0 software (free) using the Design, SurvIDINRI (for NRIs) and Multivariate Imputation by Chained Equations packages ( http://www.multiple-imputation.com/). P-values were two-sided and variables with p<0.05 were considered significantly associated with OS in multivariate models.

## Results

### Patient characteristics

The patient baseline characteristics are summarized in Table 1, most of them were male (59%) and 43% were over the age of 65. Synchronous metastasis was predominant (68%) and most of the patients with metachronous metastasis received adjuvant chemotherapy (66%, 130/196).

**Table 1:** Baseline demographic, clinical and laboratory variables for patients with and without available QoL data

		**All patients**		**Available QoL**		**Missing QoL**		**All patients**
**Variable**	Class	N	%	N	%	N	%	P
**Age**	≤65	353	57	138	55	215	58	
	>65	267	43	111	45	156	42	0.2900
**Gender**	Male	367	59	151	61	216	58	
	Female	252	41	98	39	154	42	0.5739
**PS**	0	333	54	122	49	211	57	
	1	239	38	110	44	129	35	
	2	48	8	17	7	31	8	0.0611
**Number of sites**	1	354	58	147	59	207	57	
	>1	260	42	102	41	158	43	0.5672
**Liver involvement**	No	149	24	52	21	97	27	
	Yes	460	76	197	79	263	73	0.0872
**Metastases**	Synchronous	415	68	168	68	247	68	
	Metachronous	196	32	80	32	116	32	0.9374
**Adjuvant chemotherapy**	No	488	79	200	81	288	78	
	Yes	130	21	48	19	82	22	0.4013
**Tumour site**	Colon	398	64	160	64	238	64	
	Rectum	211	34	86	35	125	35	
	both	11	2	3	1	8	1	0.6730
**LDH**	≤1xULN	380	61	134	56	246	66	
	>1xULN	240	39	115	44	125	34	0.0017
**ALP**	≤1xULN	350	56	129	52	221	60	
	>1xULN	270	44	120	48	150	40	0.0560
**CEA**	≤1xULN	177	28	61	25	116	31	
	>1xULN	443	72	188	75	255	69	0.0673
**EuroQoL**								
**Mobility**	1	223	81	223	81			
	2-3	54	19	54	19			
**Self-care**	1	255	93	255	93			
	2-3	19	7	19	7			
**Usual activities**	1	193	71	193	71			
	2-3	79	29	79	29			
**Pain/discomfort**	1	137	50	137	50			
	2-3	138	50	138	50			
**Anxiety/depression**	1	145	53	145	53			
	2-3	130	47	130	47			
**VAS score**				70 [10–100] ^**^				

^**^ Median (range).

ULN= Upper Limit of Normal.

VAS= visual analogue scale.

PS= performance status.

ALP= alkaline phosphatase.

LDH= serum lactate dehydrogenase.

**Figure 1:**
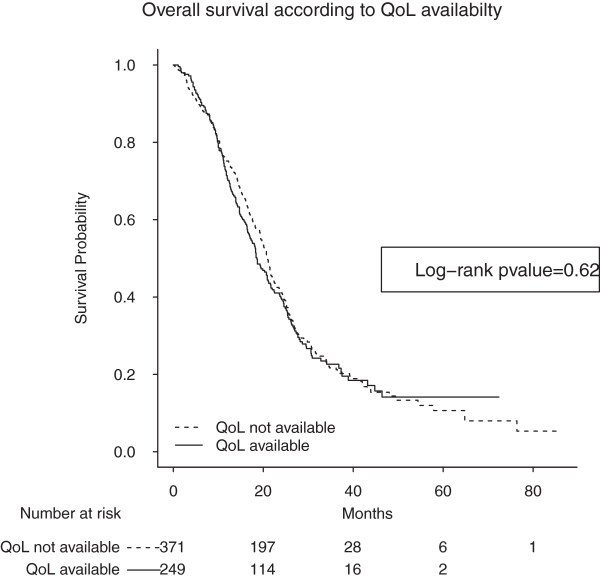
**Overall survival (in months) of patients lacking QoL data (dotted line; n = 371) and patients with available QoL data (solid line; n = 249).** Log-rank p value = 0.62. The median survival times for patient with and without QoL datasets were 18.6 months (95% CI [17.0 - 21.6]) and 20.8 months (95% CI = [19.5–22.2]), respectively.

Data on QoL was available for 249 of the 620 patients in the original OPTIMOX1 cohort (40%). Normal serum LDH was significantly more frequent in patients with missing QoL data. Patients with missing QoL data also tended to have lower serum ALP levels, a better PS and less liver involvement compared to patients with available QoL. Of the 249 patients, 75% died after a median follow-up period of 35.8 months (95% CI = [33.8–38.4]). There was no apparent correlation between the availability of QoL datasets and OS (Log-rank pvalue = 0.62; Figure 1).

Most of the patients had good QoL: 81%, 93%, 71%, 50% and 53% had no problems in terms of mobility, self-care, usual activities, pain/discomfort and anxiety/depression, respectively. The median VAS score was 70 (range = [10–100]).

### Univariate analysis

Given that”extreme problems” (coded as 3) were infrequent, QoL item scores were pooled into two classes (i.e. a score of 1 vs. a score of 2 or 3). We also combined PS into 2 classes (0 vs. 1–2), due to the low proportion of patients with a PS score of 2.

**Figure 2:**
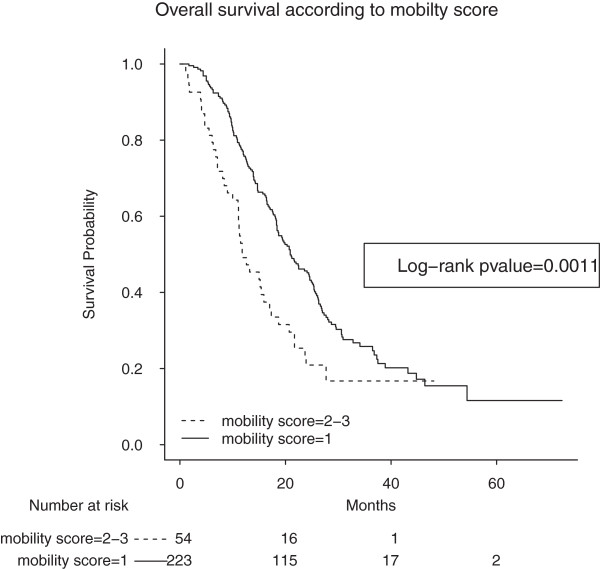
**Overall survival (in months) of patients with mobility problems (as coded 2–3) (dotted line; n = 54) and patients without mobility problems (as coded 1) (solid line; n = 223). Log-rank p value = 0.0011.** The median survival times were 20.9 (95% CI = [18.6–24.9]) months and 11.8 (95% CI = [11.1–17.3]) months for patients without problems (coded as 1) and those with problems (as coded 2–3), respectively.

Univariate analyses of clinical and QoL variables are summarized in Table 2. High serum LDH, poor PS, high serum ALP, >1 metastatic sites, age>65, high serum CEA, mobility problems (as coded 2–3) (Figure 2), pain/discomfort problems (as coded 2–3) and anxiety/depression problems (as coded 2–3) were associated with a poorer prognosis.

**Table 2:** Univariate and multivariate Cox analyses

		**Univariate analysis**			**Multivariate analysis**			**Multivariate analysis**		
					**Model not including QoL**			**Full model, including QoL**		
**Variable**	Class	HR	95% CI	P	HR	95% CI	P	HR	95% CI	P
**Age**	≤65	1								
	>65	1.42	1.06 – 1.89	0.0178						
**Gender**	Male	1								
	Female	1.06	0.79 – 1.42	0.6945						
**PS**	0	1			1			1		
	1-2	1.84	1.38 – 2.46	<0.0001	1.98	1.44 – 2.73	<0.0001	1.87	1.35 – 2.59	0.0002
**Number of sites**	1	1			1			1		
	>1	1.47	1.10 – 1.97	0.0094	1.48	1.08 – 2.05	0.0160	1.48	1.07 – 2.04	0.0176
**Liver involvement**	No	1								
	Yes	1.14	0.795 – 1.65	0.4699						
**Metastases**	Synchronous	1								
	Metachronous	0.89	0.61 – 1.29	0.5403						
**Adjuvant chemotherapy**	No	1								
	Yes	0.95	0.76 – 1.19	0.68						
**LDH**	≤1xULN	1			1			1		
	>1xULN	2.04	1.48 – 2.80	<0.0001	1.93	1.39 – 2.68	<0.0001	1.83	1.31 – 2.55	0.0004
**APL**	≤1xULN	1								
	>1xULN	1.60	1.20 – 2.14	0.0016						
**CEA**	≤1xULN	1								
	>1xULN	1.48	1.01 – 2.18	0.0444						
**EuroQoL**										
**Mobility**	1	1						1		
	2-3	1.90	1.33 – 2.71	0.0004				1.66	1.12 – 2.48	0.0117
**Self-care**	1	1								
	2-3	1.52	0.88 – 2.62	0.1322						
**Usual activities**	1	1								
	2-3	1.20	0.88 – 1.64	0.2553						
**Pain/discomfort**	1	1								
	2-3	1.39	1.04 – 1.86	0.0239						
**Anxiety/depression**	1	1								
	2-3	1.45	1.09 – 1.93	0.0116						
**VAS score**		1.001	0.996 – 1.005	0.7975						
**Harrell’s C index**						0.65 [0.61 – 0.69]			0.67 [0.63 – 0.71]	
						0.65*			0.66*	
**Schemper statistic**						9.32%			10.42%	

ULN = Upper Limit of Normal.

* = Optimism-corrected C-index.

There were no significant associations between the risk of death and self-care (p = 0.1322), usual activities (p = 0.2553) and the VAS score (p = 0.1280) QoL scales on the other.

### Multivariate analysis

The results for multivariate analyses are summarized on Table 2.

In the first model, high LDH, >1 metastatic sites and poor PS were associated with a shorter survival.

In the second model, high LDH, >1 metastatic sites, poor PS and mobility problems were associated with a shorter survival.

After multiple imputations, the pooled HR for mobility was 1.57 (95% CI = [1.16–2.12]) (p = 0.0043) in the model including LDH, the number of metastatic sites, PS, ALP, pain/discomfort and mobility (Additional file 1).

### Improvement of prognostic indices

In order to evaluate improvements in performance of the Köhne and GERCOR prognostic indices, we first calculated their performance in our population (Table 3).

**Table 3:** Improvement of Köhne prognostic index

**Köhne prognostic index**					
Variable	HR (95% CI)	P value	c-index	Schemper (%)	NRI (95% CI)
Köhne (2 vs. 1)	1.18 [0.96 – 1.47]	=0.1200			
Köhne (3 vs. 1)	2.66 [1.84 – 3.85]	<0.0001	0.54 [0.51 -0.57] *0.54	1.6	
**Improvement of the Köhne prognostic index with clinical and QoL factors: complete-case analysis**					
Köhne (2 vs. 1)	1.11 [0.80 – 1.55]	=0.5114			NRI at 12 months = 0.23 ([0.07; 0.46])
					NRI at 24 months = 0.31 ([0.16; 0.44])
Köhne (3 vs. 1)	2.17 [1.25 – 3.75]	=0.0056			
					NRI at 36 months = 0.27 ([0.02; 0.50])
LDH (>1ULN vs. ≤ 1ULN)	2.09 [1.53 – 2.87]	<0.0001	0.67 [0.63 -0.71]	10.8	
Mobility (2–3 vs. 1)	1.56 [1.05 – 2.32]	=0.0266	*0.66		
Pain/discomfort (2–3 vs. 1)	1.60 [1.17 – 2.18]	=0.0031			
**Improvement of the Köhne prognostic index with clinical and QoL factors after multiple imputation**					
Köhne (2 vs. 1)	1.24 [0.97 – 1.58]	=0.0780			
Köhne (3 vs. 1)	2.15 [1.43 – 3.24]	=0.0002			
LDH (>1ULN vs. ≤ 1ULN)	1.99 [1.61 – 2.46]	<0.0001	0.66 [0.59 -0.73]	8.63 [7.74 – 10.8]	
Mobility (2–3 vs. 1)	1.39 [1.06 – 1.83]	=0.0191	R = 65%		
Pain/discomfort (2–3 vs. 1)	1.67 [1.20 – 2.31]	=0.0031	R = 113%		

LDH = lactate dehydrogenase.

ULN = Upper Limit of Normal.

* = bootstrap C-index.

R = relative increase in variance due to missing data.

QoL = Quality of Life.

HR = Hazard ratio.

NRI = net reclassification improvement.

For multiple imputations, a logistic model was used: response variable = QoL scale (2–3 vs. 1) and exploratory variables were number of metastatic sites, liver involvement, WHO Performance Status, CEA, APL and LDH.

Variables considered in the imputation method (last model) were selected more than 5 times among the 10 replications of multiple imputations (see statistical method).

#### Improvement of the Köhne prognostic index

After addition of QoL and clinical variables to the Köhne prognostic index in a complete-case analysis (N = 236), high LDH, mobility and pain/discomfort problems appeared to be related to a shorter survival (Table 4). The C-index and Schemper statistic were improved while the NRIs were significantly different from zero (Table 3). A modified Köhne prognostic index was built using the above variables (Table 5).

**Table 4:** Improvement of the GERCOR prognostic index

**GERCOR prognostic index**					
Variable	HR (95% CI)	P value	c-index	Schemper (%)	NRI (95% CI)
GERCOR (2 vs. 1)	1.82 [1.43 – 2.33]	<0.0001			
GERCOR (3 vs. 1)	3.10 [2.38 – 4.05]	<0.0001	0.63 [0.61 -0.66] *0.63	6.44	
**Improvement of the GERCOR prognostic index clinical and QoL factors: complete-case analysis**					
GERCOR (2 vs. 1)	1.70 [1.14 – 2.54]	=0.0090			NRI at 12 months = 0.35 [0.06; 0.44]
GERCOR (3 vs. 1)	3.35 [2.20 – 5.10]	<0.0001	0.67 [0.63 -0.71] *0.67	11.52	NRI at 24 months = 0.27 [0.04; 0.38]
					NRI at 36 months = 0.28 [0.01; 0.45]
Mobility (2–3 vs. 1)	1.77 [1.19 – 2.62]	=0.0047			
Anxiety/depression (2–3 vs. 1)	1.41 [1.03 – 1.92]	=0.0314			
**Improvement of the GERCOR prognostic index clinical and QoL factors: multiple imputation**					
GERCOR (2 vs. 1)	1.77 [1.36 – 2.30]	<0.0001			
GERCOR (3 vs. 1)	2.49 [1.84 – 3.38]	<0.0001			
ALP (>1ULN vs. ≤ 1ULN)	1.25 [1.00 – 1.57]	=0.0480	0.67 [0.64 -0.71]	9.56 [8.76 – 11.52]	
Mobility (2–3 vs. 1)	1.42 [1.08 – 1.86]	=0.0120	R = 60%		
Pain/discomfort (2–3 vs. 1)	1.55 [1.10 – 2.20]	=0.0140	R = 138%		

LD = lactate dehydrogenase.

ULN = Upper Limit of Normal.

* = bootstrap C-index.

R = relative increase in variance due to missing data.

QoL = Quality of Life.

HR = Hazard ratio.

NRI = net reclassification improvement.

For multiple imputations, a logistic model was used: response variable=QoL scale (2–3 vs. 1) and exploratory variables were number of metastatic sites, liver involvement, WHO Performance Status, CEA, APL and LDH.

Variables considered in the imputation method (last model) were selected more than 5 times among the 10 replications of multiple imputations (see statistical method).

**Table 5:** Modified Köhne prognostic index

	**0 point**	**1 point**	**2 points**	**3 points**	**4 points**	**5 points**	**6 points**	**7 points**
Köhne	Köhne I	Köhne II						Köhne III
LDH	≤ 1ULN							>1ULN
Mobility score	1				2-3			
Pain/discomfort score	1				2-3			

The modified Köhne index varied from 0 to 22 points.

Poor prognosis: 15 to 22 points.

Intermediate prognosis: 8 to 14 points.

Good prognosis: 0 to 6 points.

**Figure 3:**
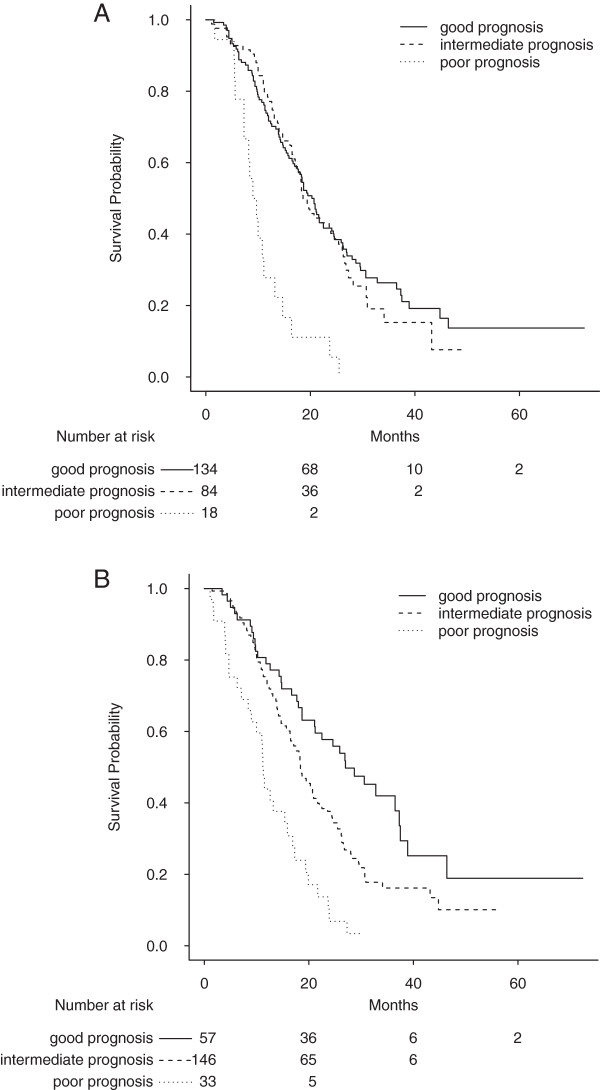
**Survival strata according to the Köhne prognostic model before and after improvement. A:** Overall survival (in months) for good, intermediate and poor prognosis according to the Köhne prognostic model. Median survival = 20.7 [17.7 – 24.4] for the group with good prognosis (n = 134); Median survival = 18.6 [17.1 – 25.4] for the group with intermediate prognosis (n = 84); Median survival = 9.0 [7.3 -14.7] for the group with poor prognosis (n = 18). Log-rank p = 0.0013. Optimism corrected C-index = 0.54. **B:** Overall survival (in months) for good, intermediate and poor prognosis according to the modified Köhne group. Median survival = 27.0 [21.1 – 37.5] for the group with good prognosis (n = 57); Median survival = 18.4 [16.5 – 21.6] for the group with intermediate prognosis (n = 146); Median survival = 11.3 [9.0 – 16.9] for the group with poor prognosis (n = 33). Log-rank p<0.0001. Optimism corrected C-index = 0.60.

Survival distributions for the Köhne and improved Köhne prognostic systems are shown in Figure 3 A&3B.

The Results of multiple imputations are summarized in Table 3.

A complete-case analysis of the GERCOR prognostic classification revealed that mobility and Anxiety/depression could improve performance: the C-index, Schemper statistic, and NRI are summarized in Table 4.

Based on these two new QoL scales, a modified GERCOR prognostic system was built using the above variables (Table 6).

**Table 6:** Modified GERCOR prognostic index

	**0 point**	**1 point**	**2 points**	**3 points**	**4 points**
GERCOR	GERCOR I		GERCOR II	GERCOR III	
Mobility score	1	2-3			
Pain/discomfort score	1	2-3			

The modified GERCOR index varied from 0 to 5 points.

Poor prognosis: 4 to 5 points.

Intermediate prognosis: 2 or 3 points.

Good prognosis: 0 or 1 point.

**Figure 4:**
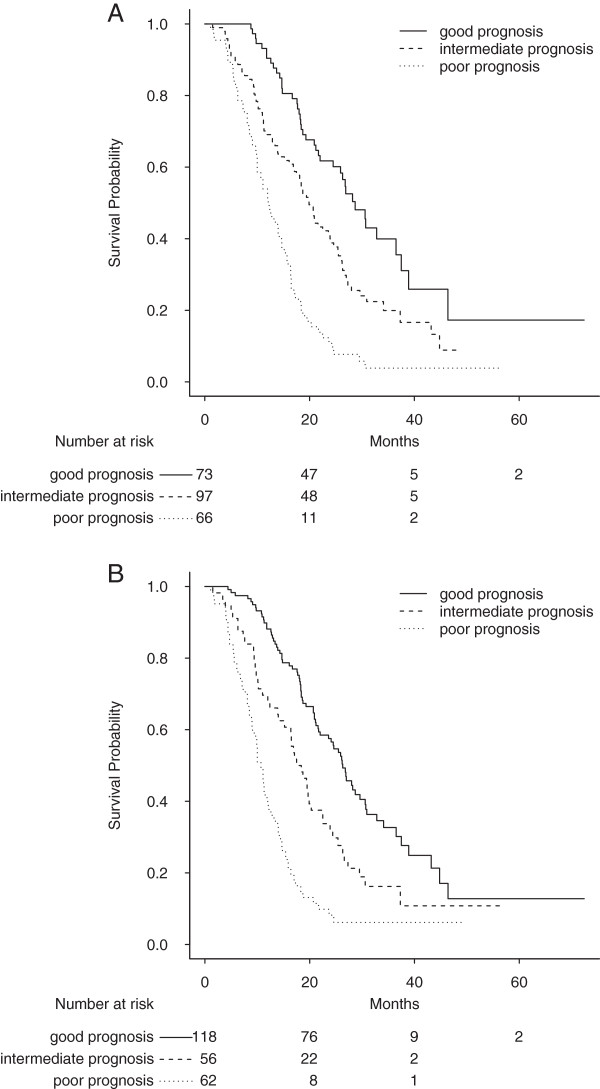
**Survival strata according to the GERCOR prognostic model before and after improvement. A:** Overall survival (in months) for good, intermediate and poor prognosis according to the GERCOR prognostic system. Median survival = 28.7 [24.5 – 38.9] for the group with good prognosis (n = 73); Median survival = 19.9 [18.1 – 23.9] for the group with intermediate prognosis (n = 97); Median survival = 12.1 [10.0 – 15.4] for the group with poor prognosis (n = 66). Log-rank p<0.0001. Optimism corrected C-index = 0.65. **B:** Overall survival (in months) for good, intermediate and poor prognosis according to the modified GERCOR prognostic system. Median survival = 28.2 [24.5 – 37.5] for the group with good prognosis (n = 68); Median survival = 21.6 [18.7 – 26.2] for the group with intermediate prognosis (n = 90); Median survival = 11.5 [10.0 – 14.7] for the group with poor prognosis (n = 78). Log-rank p<0.0001. Optimism corrected C-index = 0.66.

Survival distributions for the GERCOR and improved GERCOR prognostic systems are shown in Figure 4A and Figure 4B.

The Results of multiple imputations are summarized in Table 4.

## Discussion

In this study, EuroQol mobility dimension appeared to be the third most important prognostic factor (measured by the hazard ratio) for overall survival in unresectable mCRC, after serum LDH level and ECOG performance status. Self-reported QoL is known to be associated with OS in several types of cancer [8,9,11,12]. Our present results confirmed the independent prognostic value of QoL scales in patients with mCRC [8,11,12]. Our first multivariate model (including clinical and biochemical variables) revealed the prognostic value of LDH, PS and the number of metastatic sites, whereas our second model (with the addition of QoL) confirmed the value of LDH, PS and the number of metastatic sites and further identified the QoL”mobility" scale as an independent prognostic factor.

After multiple imputations, the mobility QoL scale remained significant despite its high associated relative increase in variance due to missing data imputation. Pain/discomfort was not significant but showed a prognostic value after the multiple- imputation analysis; this may be partially related to the high increase in variance due to missing QoL data.

We found that the Köhne prognostic system could be improved by including LDH, mobility and pain/discomfort in both complete-case and imputation analyses. Moreover, the GERCOR prognostic index was improved by mobility and anxiety/depression in a complete-case analysis and by ALP, mobility and pain/discomfort after multiple imputations. This difference in the selection of variables may be due to lack of power in the complete-case analysis albeit ALP was at the limit of statistical significance. Therefore the GERCOR prognostic index was essentially improved by QoL scales. The added value of QoL scales (completed by the patient) for improvements of the two prognostic systems revealed that the patient’s perception of his/her disease was an important information to record for prognosis assessment in addition to the clinician’s evaluation [23].

Despite a marked increase in variance due to missing data, the mobility and pain/discomfort QoL dimensions significantly improved the Köhne and GERCOR staging systems. This result comforted the independent prognostic value of these QoL scales in mCRC patients. The results for complete-case and multiple-imputation analysis were very similar. QoL significantly improved the prognostic indices with both methods (complete-case and multiple-imputation analyses). This may be related to the fact that the compete-case analysis was not biased. In fact, patients with and without QoL data at inclusion did not differ in terms of the median survival time [24] (i.e. missingness was not related to outcome).

It should be noted that such a large improvement in the C-index from 0.54 to 0.66 for the Köhne prognostic index has rarely been reported in prognostic studies. After the addition of both clinical and QoL factors, the NRIs were also statistically significant for both the Köhne and the GERCOR prognostic systems (95% CIs did not contained zero). The independent prognostic value of mobility and pain/discomfort QoL scales (using the EQ-5D) for mCRC is compatible with the result of Efficace [12] regarding the prognostic value of social functioning scale (using the EORTC QLQ-C30). In fact, mobility and pain problems could impair the social functioning QoL dimension.

One of the present study's strengths relates to its use of the easily understood and rapidly completed EQ-5D. The EQ-5D was chosen because it was expected to be less time consuming and could prevent missing data. However, EQ-5D is not a cancer-specific questionnaire like the EORTC QLQ-C30 and it constitutes a limitation of our study. The high proportion of missing data (60%) and its large variability between countries (ranged from 5% to 66%) constitute another limitation in the generalizability of our results. Such a large heterogeneity in missing data might be related to the trial logistic and/or each country’s culture. It is also important to note than our population came from a randomized controlled trial with restrictive inclusion and non inclusion criteria and might not be representative of mCRC patients in general [25]. Quality of Life may be an important parameter to record when assessing the situation of mCRC patients, since it improved the accuracy of OS prediction and greatly improved the two best-known prognostic classification systems for mCRC. We consider that QoL domains are important factors in the field of stratified therapy in the sense that knowing some aspect of the patient’s self-reported QoL level could be decisive in the choice of different treatment options in the area of tailored medicine. By way of an example, a clinician might wish to avoid a treatment with pain as side-effect if the patient reported preexisting pain symptoms. Pain and mobility could also serve as an inclusion and/or stratification factor in randomized, controlled trials in mCRC.

## Conclusion

Our results confirmed the prognostic value of QoL in mCRC patients. Thus, QoL scores should be recorded as it could give supplementary information to the clinician regarding the prognosis of a patient as well as in the judgment of an acceptable treatment side effect.

## Abbreviations

QoL: Quality of life; mCRC: Metastatic colorectal cancer; OS: Overall survival; EQ-5D: Generic measure of health status developed by the EuroQol Group; HR: Hazard ratio; CI: Confidence interval; CRC: Colorectal cancer; TNM: Tumor Node Metastasis; GERCOR: Groupe Coopérateur Multidisciplinaire en Oncologie; EORTC: European Organization for Research and Treatment of Cancer; VAS: Visual analogue scale; PS: Performance status; ALP: Alkaline phosphatase; LDH: Lactate dehydrogenase; ITT: Intention to treat; NRI: Net reclassification improvement; CEA: Carcinoembryonic antigen; HDL: High density lipoprotein.

## Competing interests

The authors declare that they have no competing interests.

## Authors’ contributions

MD, BC, FB, AG, CL, CT, TF, NP, SD, AH, MH and MG the seven authors are justifiably credited with authorship, according to the authorship criteria. In detail: MD BC TF FB – conception, design, analysis and interpretation of data, drafting of the manuscript, final approval given; AG CL CT SD NP AH MH MG BC – acquisition of data, interpretation of data, critical revision of manuscript, final approval given. All authors read and approved the final manuscript.

## Acknowledgements

The authors thank Dr. David Fraser for advice in English language.

## Supplementary Material

Additional file 1 Results of the multivariate analysis after QoL imputation. Click here for file
