# Supplementary material for: Could baseline health-related quality of life (QoL) predict overall survival in metastatic colorectal cancer? The results of the GERCOR OPTIMOX 1 study
Source: Health Qual Life Outcomes. 2014 May 13;12:69. doi: 10.1186/1477-7525-12-69 (PMC4029890; doi:10.1186/1477-7525-12-69)
Supplement: Additional file 1 — Results of the multivariate analysis after QoL imputation. [file 1477-7525-12-69-S1.doc]

**Additional file 1: Table S1: Results of the multivariate analysis after QoL imputation:**

ALP=alkaline phosphatase

LDH= lactate dehydrogenase

PS=performance status

| Variable | HR (95%CI) | P value | C-index | Schemper (%) |
| --- | --- | --- | --- | --- |
| PS (coded 1-2 vs. 0) | 1.31 [1.07 – 1.61] | =0.0091 |  |  |
| LDH (>1 ULN vs. ≤ 1 ULN) | 1.76 [1.39 – 2.22] | <0.0001 |  |  |
| ALP | 1.29 [1.06 – 1.60] | =0.0216 | 0.66 [0.63 -0.70] | 9.1 [7.8 – 10.4] |
| Number of sites | 1.53 [1.26 – 1.85] | <0.0001 |  |  |
| Mobility (coded 2-3 vs. 1) | 1.57 [1.16 – 2.12] | =0.0043 | R=78% |  |
| Pain/discomfort (coded 2-3 vs. 1) | 1.28 [0.98 – 1.66] | =0.0702 | R=87% |  |

ULN=Upper Limit of Normal

R=relative increase in variance due to missing data

The above six variables were chosen because they were selected more than 5 times among the 10 replications of multiples imputations (cf. statistical method).

For multiple imputations, a logistic model was used: response variable=QoL scale (2-3 vs. 1) and exploratory variables were number of metastatic sites, liver involvement, WHO Performance Status, CEA, APL and LDH.
